# Conservation facelift: The mesoface

**DOI:** 10.1016/j.jpra.2026.07.024

**Published:** 2026-07-21

**Authors:** Pierre Quinodoz, Matteo Scampa, Lakhdar Belhaouari

**Affiliations:** a Hôpital de la Tour, CH-1217 Meyrin, Switzerland; b Department of Plastic, Reconstructive, and Aesthetic Surgery, Geneva University Hospitals, Geneva University, 1205, Geneva, Switzerland; c Centre de Chirurgie Esthétique, Toulouse, France

**Keywords:** Facelift, Deep plane, Mesoface, Cosmetic, Conservation

## Abstract

**Introduction:**

Facelift procedures maintain a central role in facial rejuvenation and require a balance between safety, efficacy and natural outcomes.

**Material and methods:**

In this article, the authors describe their approach to deep plane facelift, based on their knowledge of facial anatomy and dynamics of facial aging. They emphasize the cohesive mesoface unit comprised of skin, subcutaneous fat and the mesoface fat pad. They describe their approach to the deep plane face lift focusing on repositioning of the mesoface fat pad with limited undermining.

**Results:**

The authors performed 864 facelifts over the last 5 years with 0 nerve complications and 37 hematomas reported. The results were considered harmonious and appeared durable, with the technique tailored to each patient’s anatomy and addressing the natural descent of the face.

**Conclusion:**

The described facelift technique is a deep plane facelift tailored to each individual patient with anatomical preservation.

## Introduction

Facial rejuvenation has advanced with a focus on natural results, structural preservation, safety, and individualized techniques. The Preservation Facelift has gained recognition for minimizing surgical trauma through limited skin undermining, a more proximal SMAS entry point, and the release of targeted ligaments, which improves the vascular reliability of the composite skin flap.^1^ However, the Conservation Facelift represents a further refinement, one that is highly patient-specific, meticulously precise, and deeply conservative.^2^ Why “Conservation Facelift”?

It’s a matter of semantics: conservation means protecting nature through active management, whereas preservation means leaving nature untouched, intact in its original state.

This technique refines deep plane lifting by selectively addressing sagging beneath the SMAS with limited dissection. This technique builds upon our initial publication^3^ and is further developed in this article, with an emphasis on a dynamic approach to anatomy. Its objective is to access the deep plane between the mesoface and metaface, two vertical subdivisions of the face that exhibit distinct aging dynamics. Unlike traditional facelifts that use generalized landmarks, the Conservation facelift defines the mesoface preoperatively, facilitating more precise, natural-looking correction and long-lasting support. In contrast to the preservation facelift, the Conservation facelift limits deep dissections, ligament release, and SMAS manipulation, focusing solely on repositioning the tissues that sag without disrupting them.

This article outlines the authors approach to deep plane facelift based on their own anatomic concept with critical structures preservation and understanding of dynamic facial aging.

## Materials and methods

### Anatomical, dynamic, and clinical foundations of the conservation facelift

**Fig. 1:**
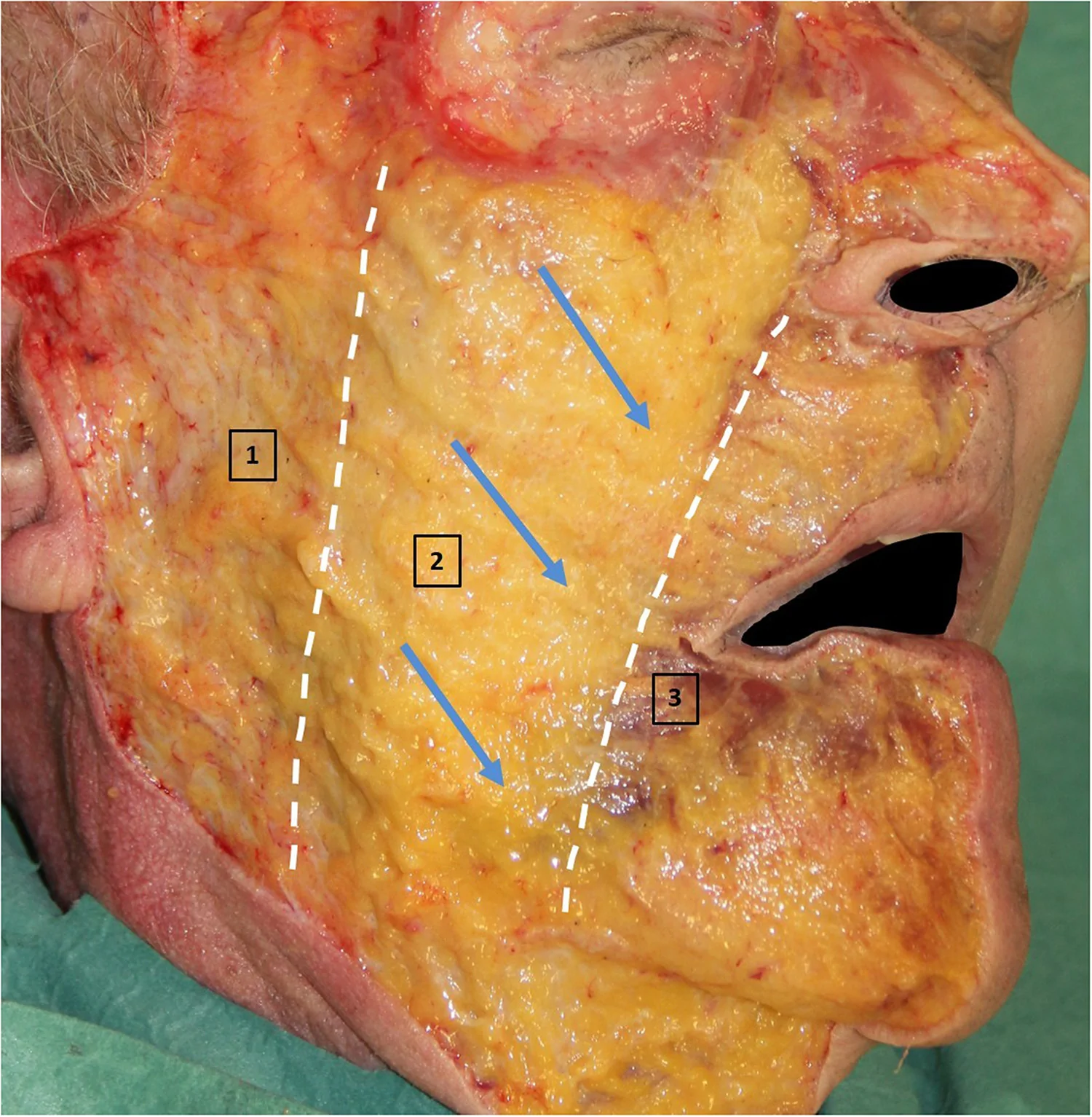
Fresh cadaver dissection describing the vertical division in thirds. 1.Metaface 2. Mesoface 3. Proface. Arrows show the vector of mesoface sagging.

This technique is based on anatomical and dynamic principles of facial aging, with specific attention to changes occurring over time in the mesoface.^4^^,^^5^ Previous work has divided the face vertically into three zones: the metaface (posterior), mesoface (central), and proface (medial).^4^^,^^5^ (Fig. 1) Unlike the horizontally defined midface, which includes the cheeks, nasolabial folds, and peri‑orbital area but excludes the jawline, the mesoface provides a more comprehensive view of aging. The mesoface represents a conceptual reinterpretation of facial structures, considering the superficial malar fat pad and its jowl extension as a single anatomical unit together with the overlying skin, rather than distinct fat compartments. It contains multiple fat compartments, retaining ligaments, and mimetic muscles that influence facial expression. This subdivision is derived from clinical observation and anatomical dissections, which revealed that, with aging, the mesoface progressively descends in a downward and medial vection, eventually extending below the jawline, whereas the proface and metaface remain relatively stable. This vertical displacement contributes to the loss of jawline definition and the development of jowls, a central concern in facelift surgery. By understanding this distinction, surgeons can tailor their approach to target the natural gravitational descent of the face.

### Anatomical planes involved at the mesoface level

**Fig. 2:**
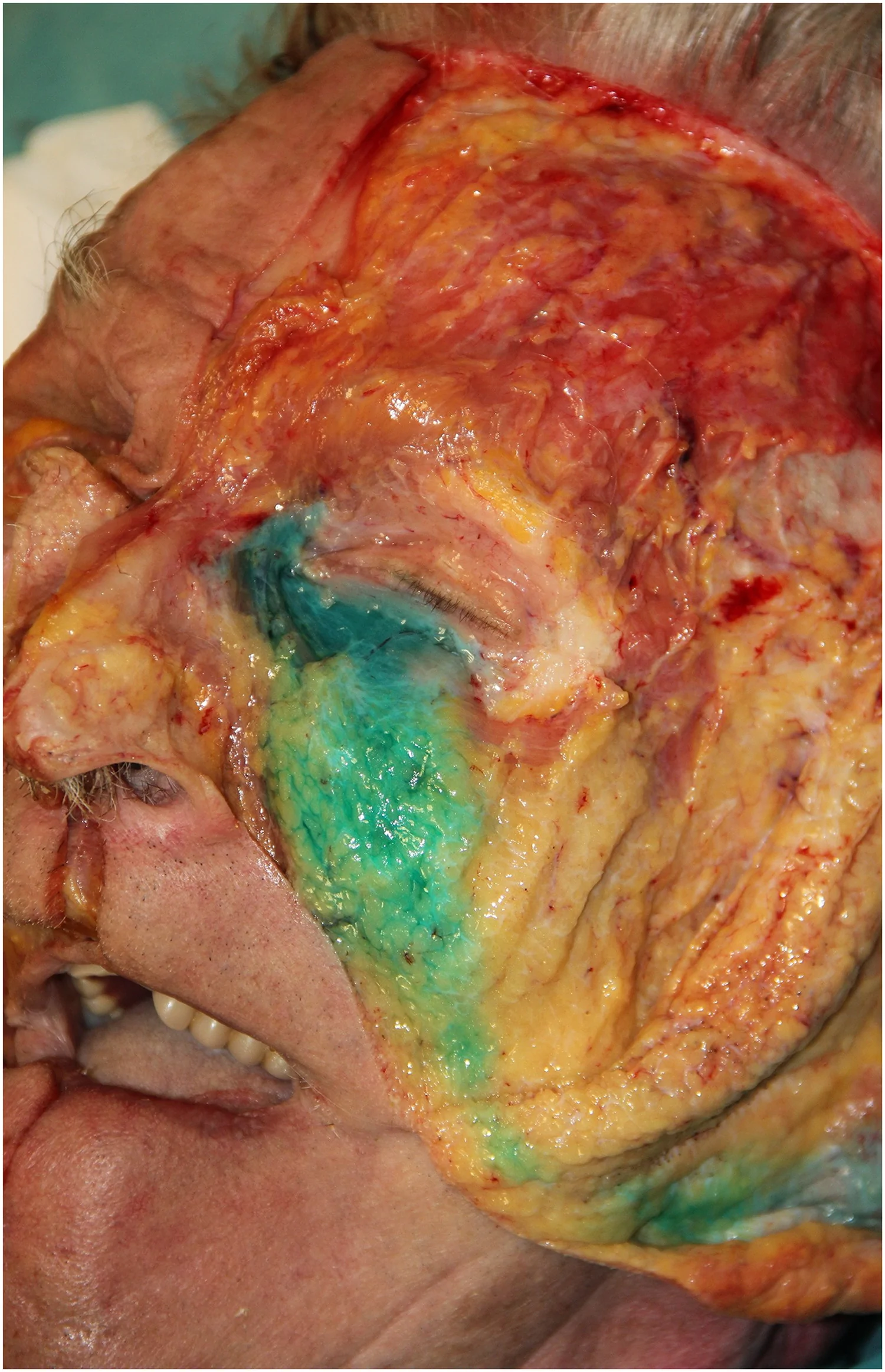
Fresh cadaver dissection with injection of blue dye in the mesoface. Note how the blue dye remains limited to the mesoface unit and diffuse toward the jowl.
- Muscular Plane: Specifically targeting the Orbicularis Oculi and Platysma muscles, which may lose tone with age.
- Deep Malar Fat Compartment: Firmly attached to the malar bone, this area and Bichat’s fat pad remain non-mobile and are preserved to maintain functional integrity, as they do not sag.
- Underlying Bone Structure: The bone structure provides essential soft tissues support and serves as a stable anchor for repositioning efforts.

Our technique addresses specific anatomical layers in the mesoface, including:

- Skin and Subcutaneous Fat Layer: The outermost layer affected by age-related ptosis.
- Fat Compartment Unit: The mesoface including the superficial malar fat pad and its extension toward the jowls and sometimes below the jawline in the upper neck.^4^^,^^5^ This compartment can be considered as a single continuous unit from the midface to the jowls. We define it as an anatomical unit, a functional unit in facial expression and a single unit in facial aging process. (Fig. 2) The midfacial subdivision previously described by Rohrich was not reproducible in our facial dissections and our finding described and published in 2017 5 are compatible with a recent article by Minelli.^6^^,^^7^ When methylene blue (1% concentration, diluted 1:5 in saline) was injected with a cannula in the cranial part of the malar fat pad in fresh cadavers, dye diffusion in the jowl was observed during dissection.

### Dynamic considerations

Facial expression and age-related sagging primarily affect the skin, subcutaneous layer, and superficial fat of the mesoface. Although muscle tone decreases with age and contributes to facial ptosis, its impact is negligible. In contrast to Val Lambros' description, we believe the palpebro-malar groove is not a fixed structure.^8^ While the bone and deep malar fat remain stationary, the orbicularis oculi muscle, superficial malar fat, and overlying skin glide over these deeper fixed structures during facial expressions and age-related sagging. Facial sagging mainly affects the mesoface, while aging in the metaface and proface is less pronounced.

**Fig. 3:**
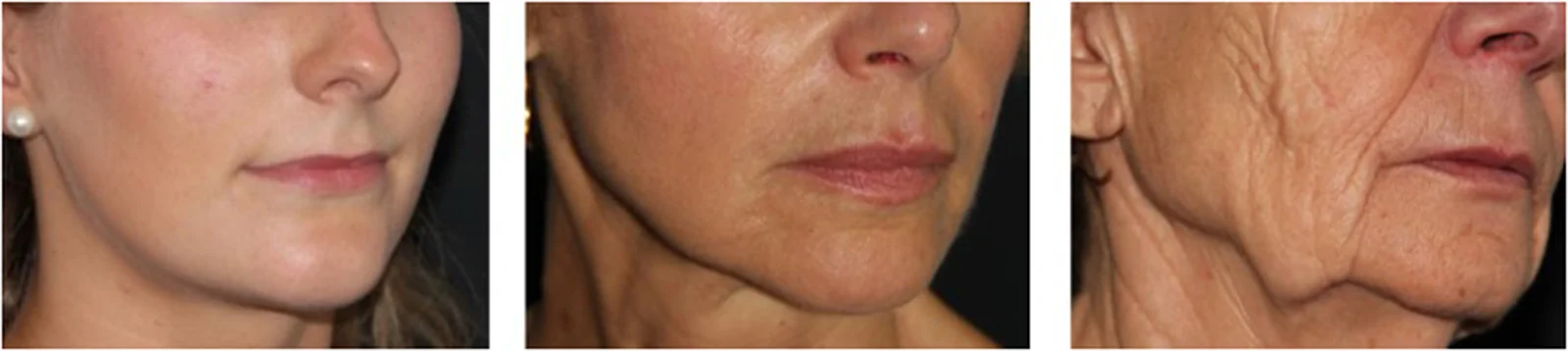
Clinical pictures depicting the progressive sagging of the mesoface at 3 different ages: young, mature and elderly.

The mesofacial fat compartment is an anatomical and dynamic unit, extending from the midface superficial malar fat to its lower face continuation, including the jowls. With aging, this fatty compartment descends, pulling the overlying skin downward and contributing to the characteristic appearance of facial sagging. (Fig. 1, Fig. 3). This natural descent is facilitated by the deep fascial layer, which allows three-dimensional tissue gliding through multiple deep glide planes separated by retaining ligaments, consistent with the anatomical observations reported by Minelli et al.^9^ Clinical signs of mesofacial aging include unwinding of the nasolabial fold, the appearance of jowls with a prejowl sulcus, and extension of the mediojugal groove from the tear trough, accompanied by palpebro-malar groove deepening and descent.

**Fig. 4:**
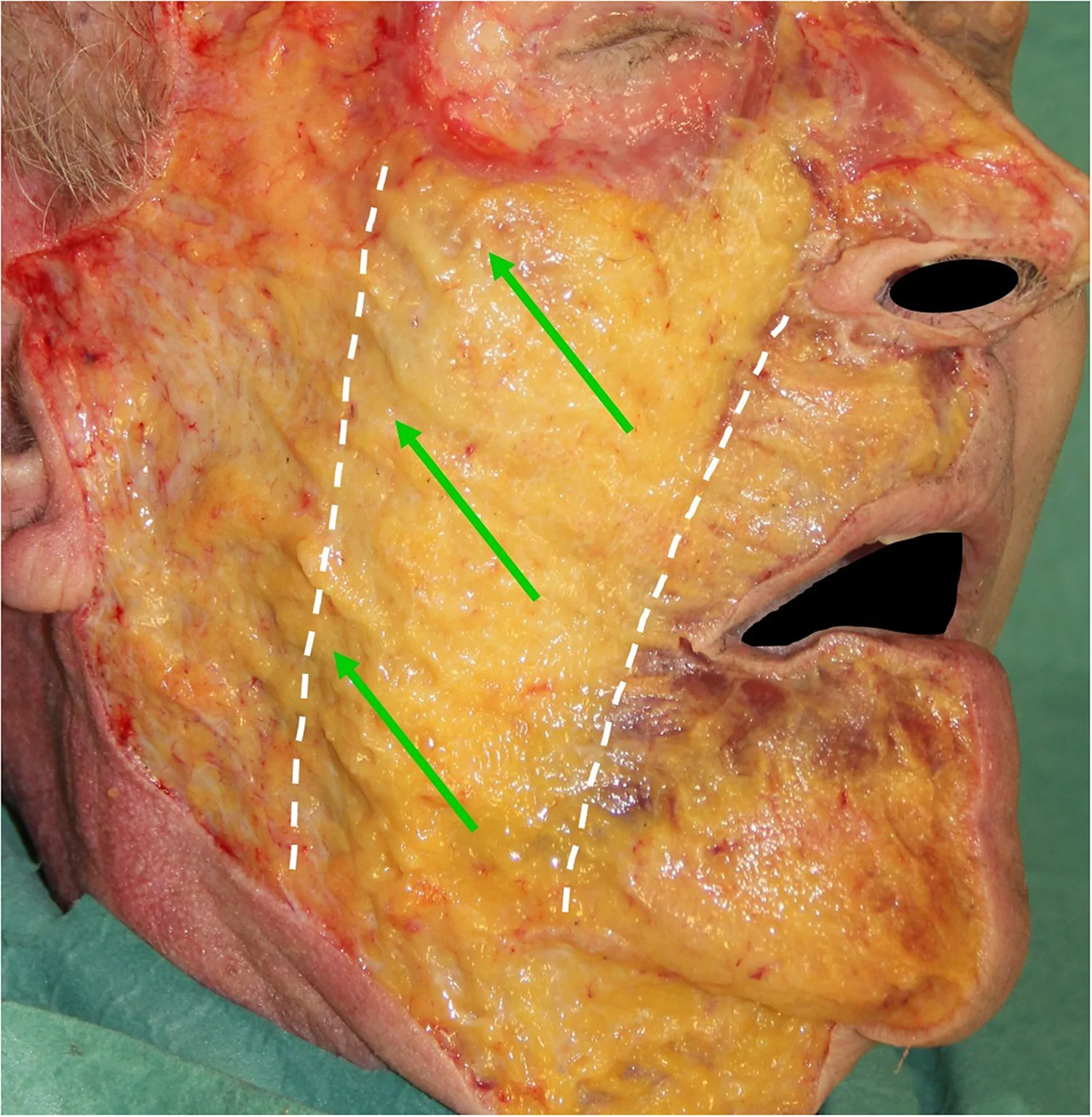
Fresh cadaver dissection demonstrating the vector required for repositioning of the mesoface.

**Fig. 5:**
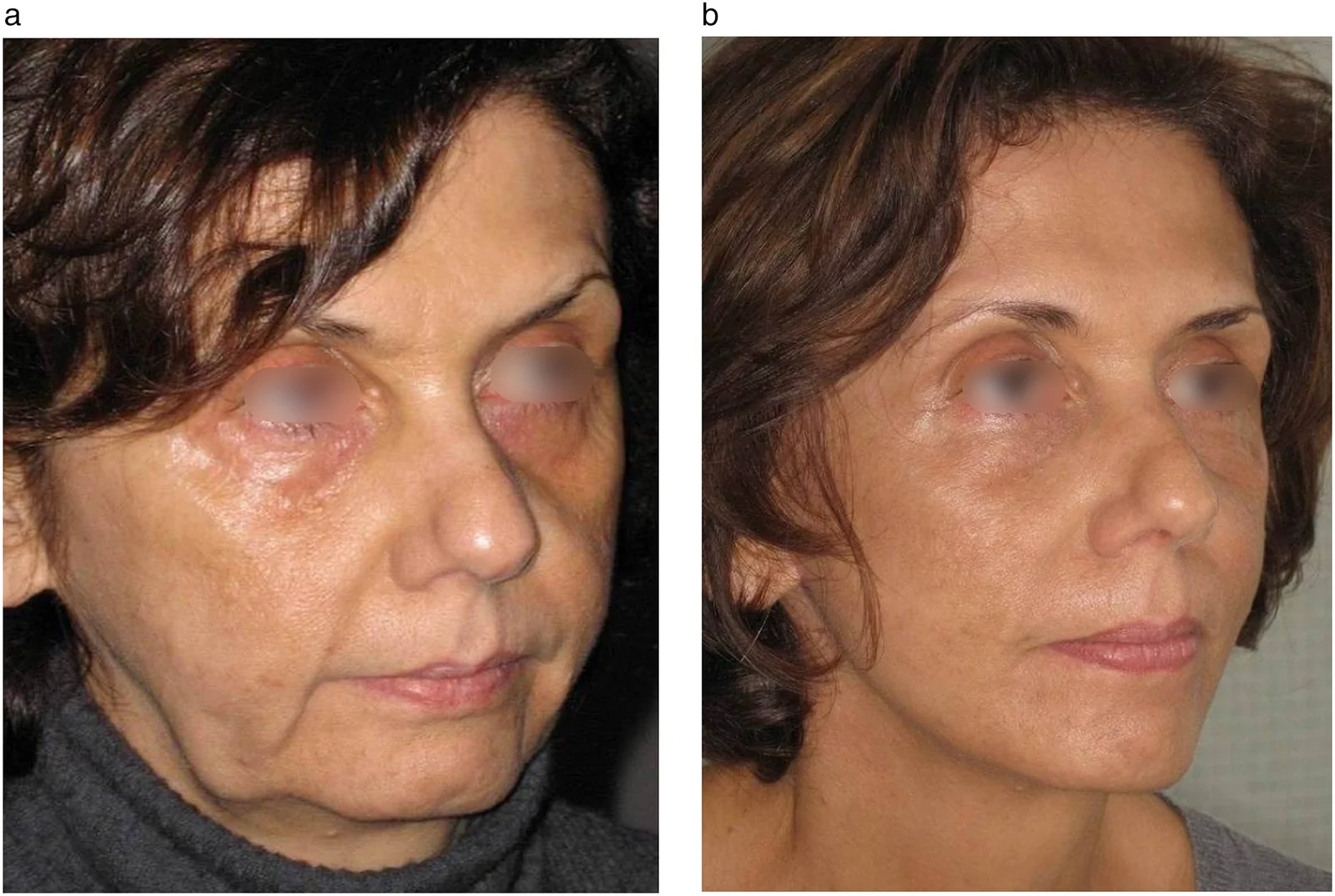
Surgical outcomes of our Conservation facelift technique in a 65 years old patient. Note how repositionning the mesoface addresses the jowl sagging. a: pre-operative. b: 1 year post-operatively. The patient benefited from a necklift as well.

We focus on the layers prone to sagging: skin, subcutaneous fat, and superficial mesofacial fat pad, thereby avoiding unnecessary intervention on deeper structures and promoting natural results.^5^ (Fig. 4, Fig. 5).

### Conservation facelift: Our technique

One essential step is pre-operative markings. Most facelift techniques are based on static anatomical landmarks and often adopt a standardized ("one-size-fits-all") approach. However, every face is unique, as each patient has his own unique mesoface. It is essential to assess the patient in an upright position and precisely trace the mesoface contour, including the sub-orbital region and jowl area. In thin patients, a shadow zone appears between the mesoface and metaface in the standing position. When facial contours are not easily visible, palpation can determine the dividing line between the mesoface and metaface. The contour is easily determined in patients for whom the indication is justified by a sagging jowl and a body mass index (BMI) close to the norm.

**Fig. 6:**
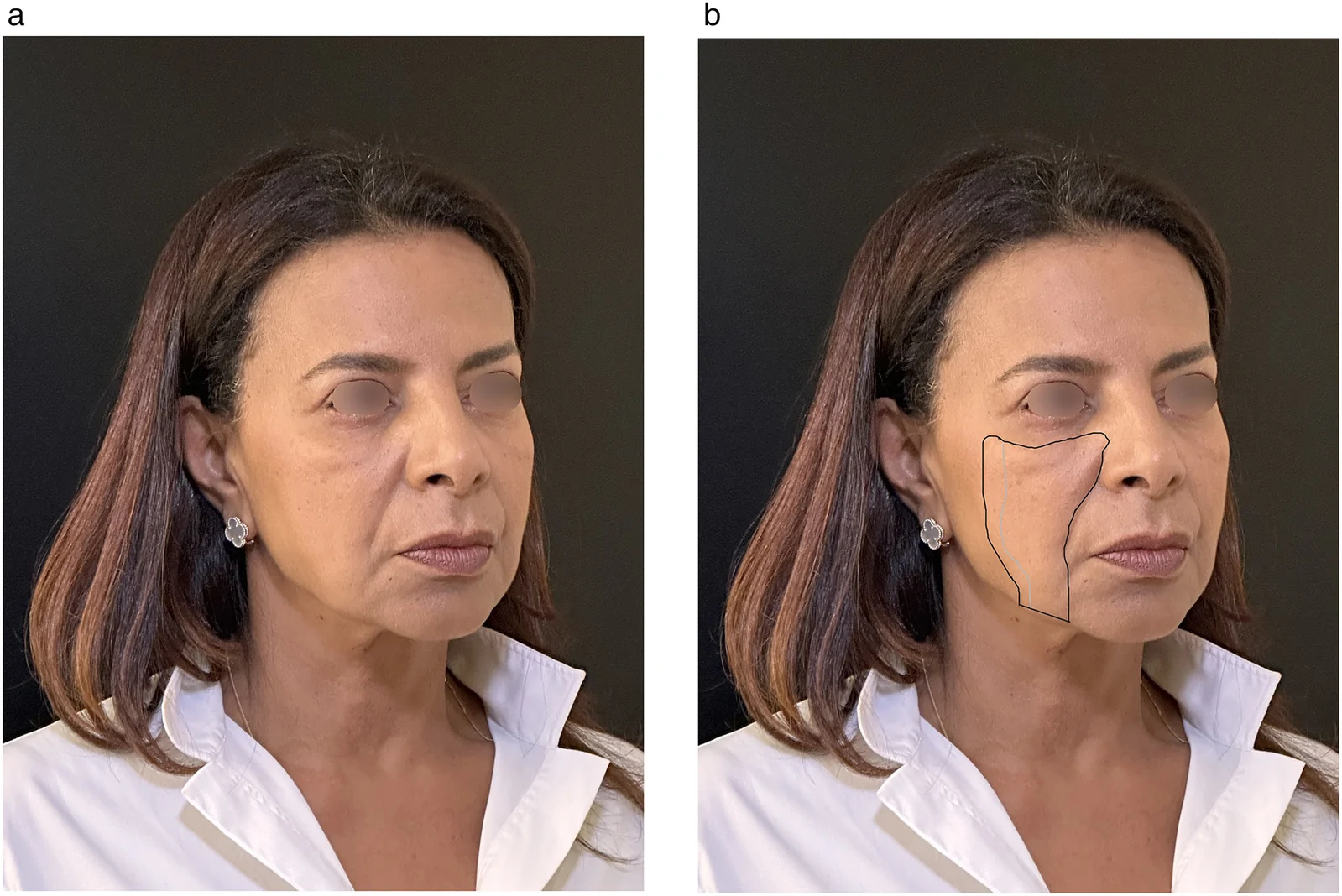
Surgical drawings for our technique. a. Patient without drawings, note the sagging of the mesoface including the jowls. A subtle shadow defines the lateral contour of the mesoface.b. Black line: Demarcation of the mesoface unit. Grey line: dissection extent above and below the mesoface fat pad.

We then identify fixation points needed to place anchors sutures that will lift the mesoface harmoniously. The deep plane entry point will be the posterior free edge of the mesoface superficial fat pad. (Fig. 6a–c).

**Fig. 7:**
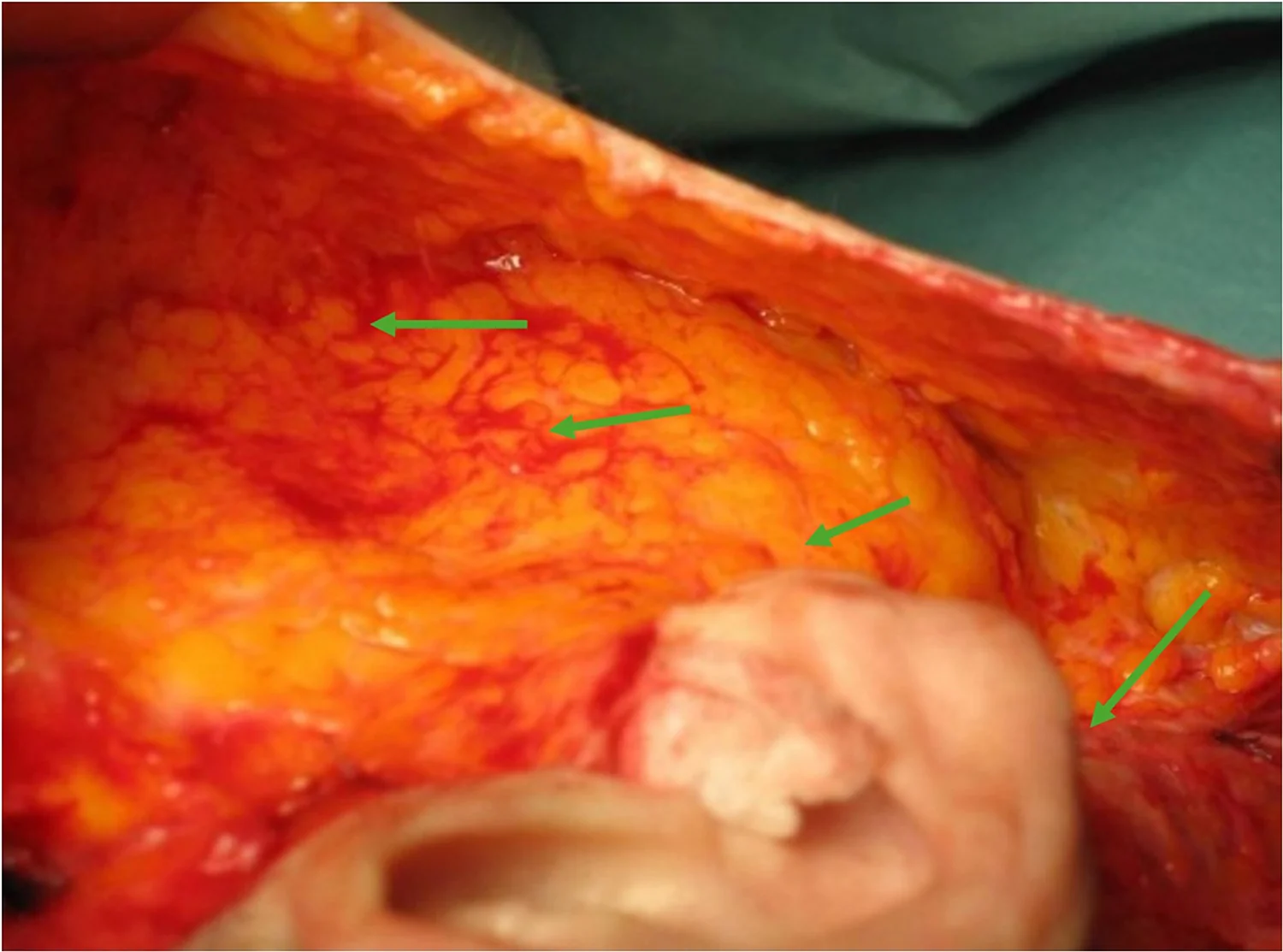
Per-operative surgical dissection of the right cheek with exposure of the mesoface superficial fat pad. Green arrows demonstrate the repositioning vectors for the Conservation facelift.

**Fig. 8:**
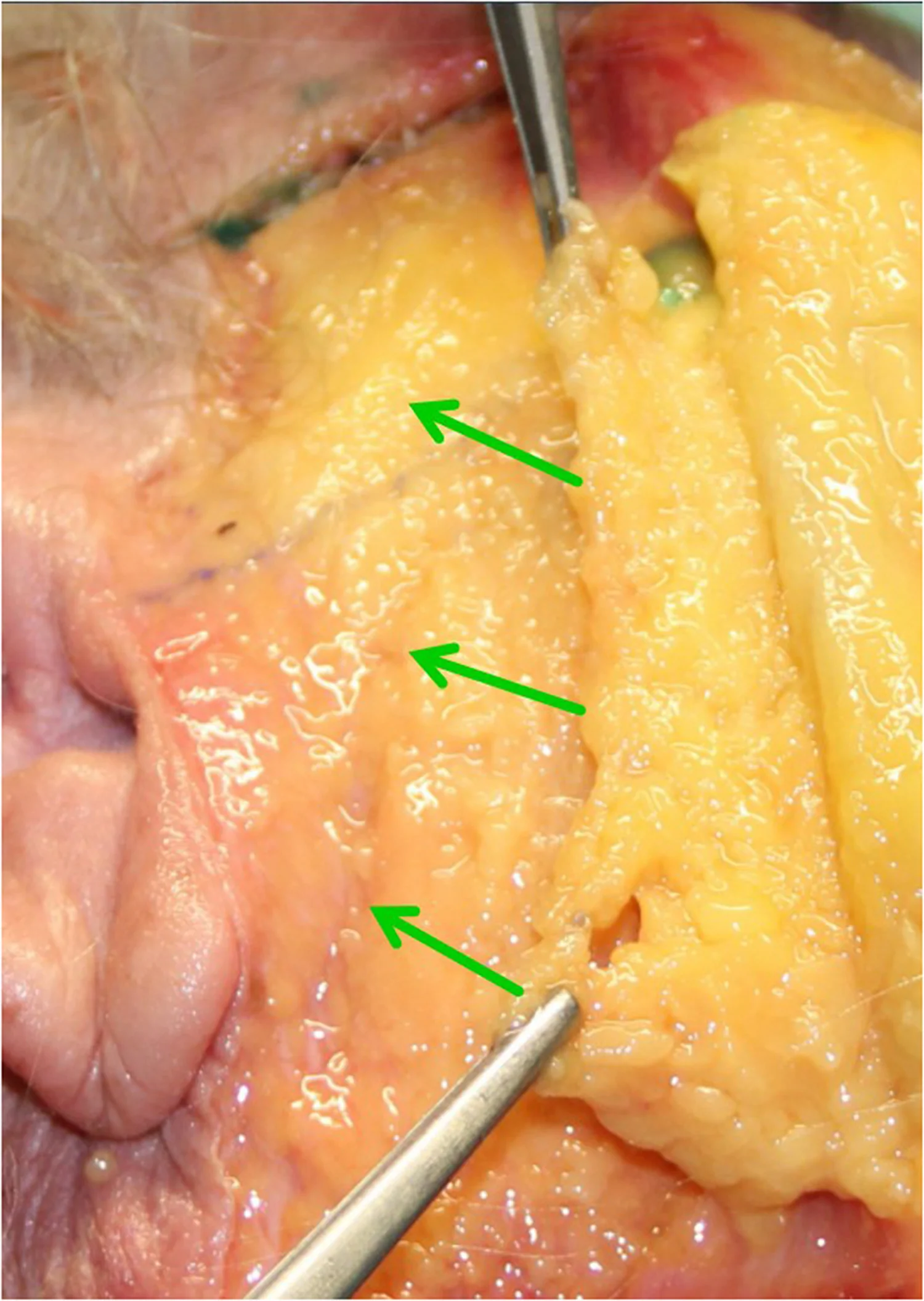
Fresh cadaver dissection demonstrating mesoface repositionning as a single unit. Green arrows indicates the vector of repositionning.

**Fig. 9:**
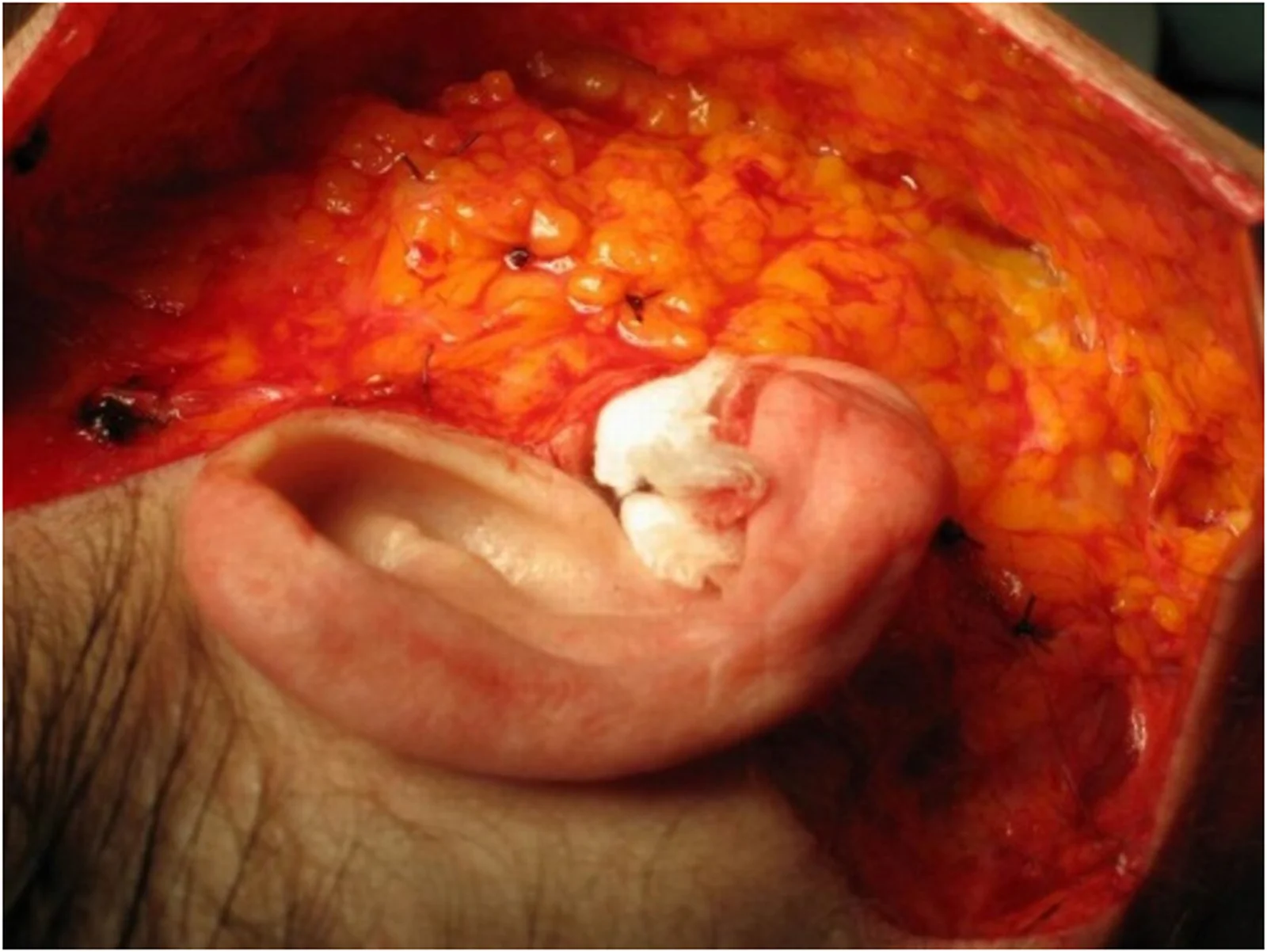
Per-operative surgical dissection demonstrating the repositionning of the mesoface following the vectors previously described.

We begin with subcutaneous dissection in the cheek area of the metaface, extending conservatively over the mesoface fat pad by 2–3 cm, exposing the superficial fat compartment of the mesoface (Fig. 7). Undermining should not be extensive, especially near the nasolabial fold, because the skin and fatty mesoface (including superficial malar fat and jowl fat) form an anatomically cohesive unit that moves, ages, and descends together, requiring repositioning as a single unit. (Fig. 8, Fig. 9).

The previous anatomical description shows us 4 possible dissection planes through the 5 described layers:

- A superficial plane between the skin and the mesoface fat that can be described as superficial plane.
- A deep plane between the bone and deep malar fat pad, strongly attached to the bony structure of the face, described in the subperiosteal Mask lift by Tessier.^10^
- A deep plane between the deep malar fat and the muscles that contains high nerve density and key structures such as Stensen’s duct and facial vessels.
- A deep plane between the muscles and the fatty mesoface, used in our technique.

**Fig. 10:**
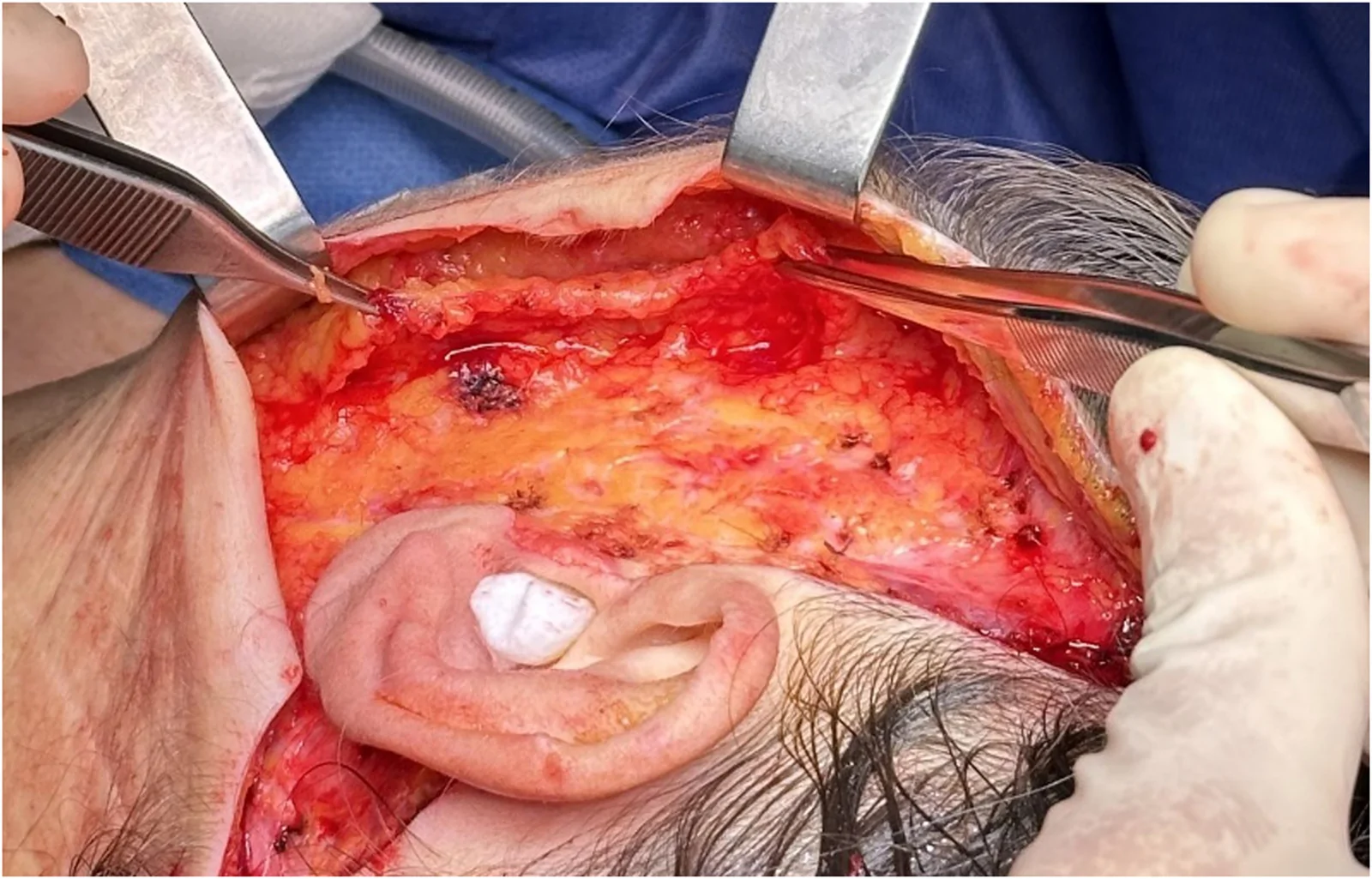
Per-operative view of the surgical dissection under the mesoface. Please note the limited dissection in the deep plane, 1 to 2 cm are deemed sufficient by the authors.

**Fig. 11:**
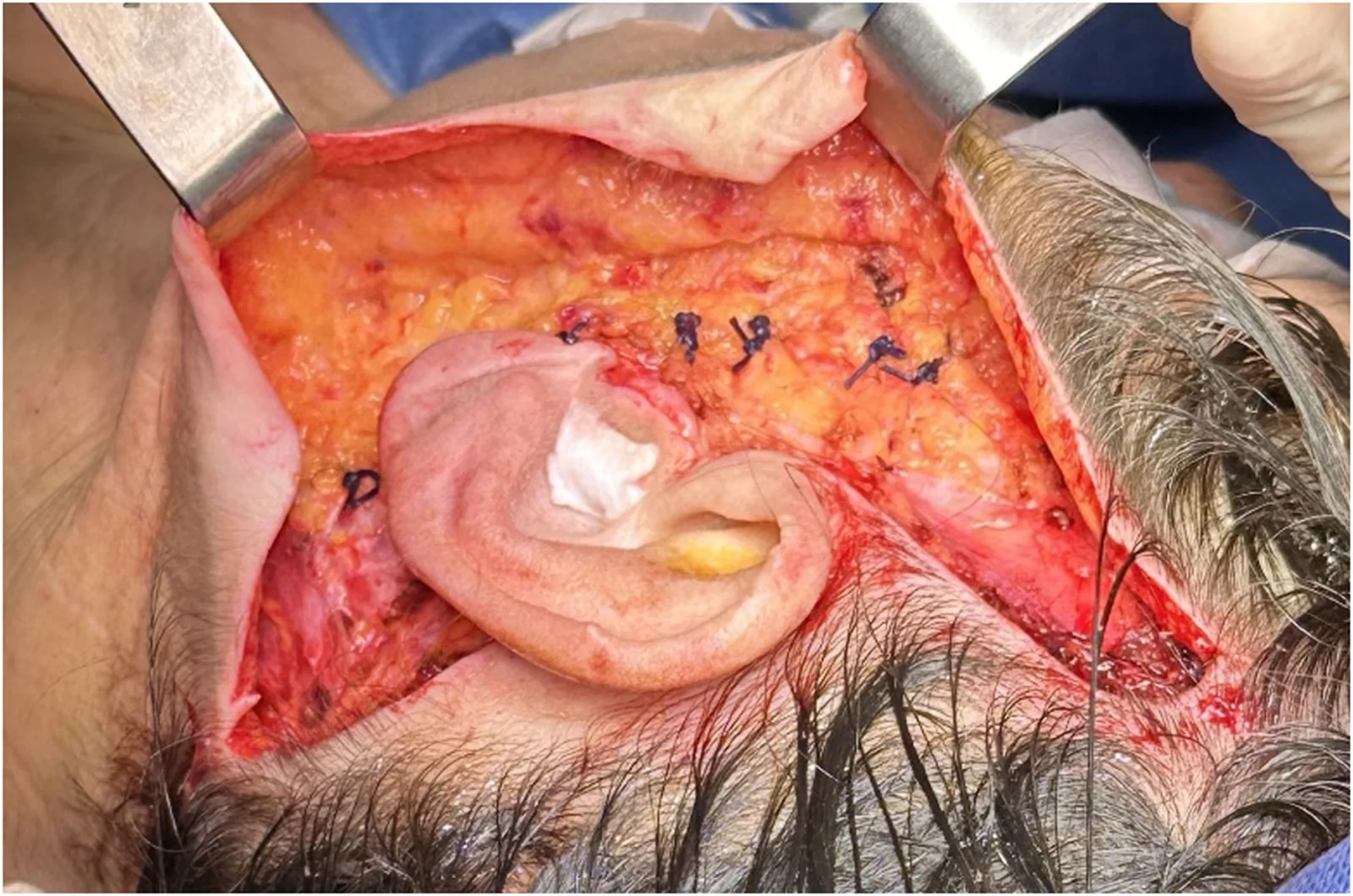
Per-operative view of mesoface fixation after the deep plane undermining described in Fig. 10. Fixation is achieved by strong breaded sutures such as Vycril #1. - All illustrations copyright Lakhdar Belhaouari and Pierre Quinodoz.

Sagging and dynamic expression are largely limited to the layer beneath the fatty mesoface, extending from midface to jowls. Lifting within this plane allows for precise repositioning and secure tensioning of the fatty mesoface, as natural attachment points are leveraged for better stability, enhanced re-tensioning, and more durable results*.* A 2-cm dissection is sufficient to provide a reliable anchor for lifting the fatty mesoface and overlying skin (Fig. 10, Fig. 11). Repositioning is achieved with firm fixation using a robust, slowly resorbable suture (such as Vicryl #1, or alternatively #2 or #0), with a resorption period of at least four months to ensure durable results. (Fig. 9, Fig. 11). We prefer Vicryl over PDS, despite its larger size, it is more flexible and lasts in our experience at least as long as PDS. We stopped using non-absorbable sutures due to skin extrusion risks. In cases of very moderate ptosis, extensive undermining is unnecessary: instead, plication points may be used. We anchor the mesoface fat to the deep temporal fascia. This ensures a vertical and lateral vector, preventing an unnatural stretched look. For the jowl, we prefer a pre-tragal fixation point with anchoring to the parotid fascia and SMAS, or Lore’s fascia.

In the cheek lower portion, the platysma may be exposed, yet extensive sub-platysmal dissection is unnecessary. Our Conservation facelift technique for the lower face has a profound impact on the neck. For patients with more pronounced cervical skin excess, a slightly more vertical repositioning vector effectively addresses this issue. The Conservation facelift can be safely associated with complementary procedures such as lipofilling or blepharoplasty.

### Concept of safety

Our technique can be considered safe because:

• We reposition the structures that sag due to the aging process. We consider the platysma and fatty mesoface rather than the SMAS.
• Undermining beneath the fatty mesoface is limited to 1–2 cm, away from critical structures (buccal branches of the facial nerve, facial artery and vein). Deep plane dissection is performed selectively, based on dynamic preoperative assessment and marking. The deep plane entry point is then confirmed during the surgery, at the interface between the metaface and mesoface.
• The dissection above the fatty mesoface is limited (1 to 2 cm) because the fatty mesoface and the skin can be considered as a single unit. By lifting the fatty mesoface, we lift jowl skin and unfold the naso-labial fold. This limited dissection results in less vascular disruption, thereby reducing the risk of hematoma.
• Platysma dissection in the region of the horizontal branch of the mandible is minimal (approximatively 2 mm to anchor suspension sutures), therefore not exposing the surgeon to facial nerve or submandibular glands lesions.
• Our technique reduces the risk of injury to critical structures and suspension ligaments by targeting only sagging tissues and minimizing deep-plane dissection.

These elements and concepts allow a safe approach to the deep plane facelift.

### An anatomically respectful technique

Our technique is a conservative procedure inspired by the natural dynamics of age-related descent. This technique retains the core principles of the preservation facelift, such as limited skin dissection, natural results, and avoiding disruption of retaining ligaments.^2^^,^^11^ It focuses on identifying the mesofacial border and repositioning only the tissues affected by sagging, therefore we prefer the term Conservation facelift.

## Results

The two senior authors (P.Q. & L.B.) performed 864 facelifts using this method over five years. No nerve complications (definitive or transient nerve paresis) were reported, evaluation was performed by clinical examination immediately after surgery and at each scheduled postoperative follow-up visit. Whenever there was uncertainty regarding possible weakness, postoperative facial movements were compared with the patient's preoperative video recordings to assess facial symmetry and range of motion. A total of 37 hematomas were recorded, all were easily managed on an outpatient basis. Drainage involved reopening the surgical approach under analgo-sedation, evacuating the blood clot, hemostasis, and saline irrigation. In our experience, these complications did not significantly affect postoperative recovery. No skin necrosis was reported. Results appear stable over time, and the few cases of skin slackening observed were mainly linked to postoperative weight loss. Since then, we have systematically assessed all our patients before the day of surgery, giving preference to those whose BMI is close to the norm.

## Discussion

### Reevaluating the concept of the midface and fat compartments in facial anatomy

In the ongoing study of facial anatomy, there is ongoing debate over the precise understanding of structural compartments and terminology.^9^ Minnelli challenges the idea of distinct fat compartments in the midface region and has argued that the concept is anatomically unfounded.^7^ This assertion supports our previous findings on the concept of Mesoface published in French language in 2017^4^^,^^5^; following dissection of >150 fresh specimen heads, marked by methylene blue injections failing to demonstrate distinct compartments within the superficial malar fat.

#### The concept of fat compartments in the midface region

Rohrich and colleagues, through cadaveric dissections, described distinct facial fat compartments, identifying three separate units in the malar region: the medial, middle, and lateral temporal-cheek fat compartments.^6^ This concept of distinct fat compartments in the midface was later challenged by Minelli, who argued that the facial fat layers form a continuous structure rather than being strictly compartmentalized.^7^ This understanding is important for refining anatomical descriptions and guiding surgical interventions. Our Conservation facelift aligns with Minnelli’s emphasis on anatomical continuity, focusing on repositioning lax skin and subcutaneous layers with minimal disruption to deep structures. By acknowledging the integrated nature of facial fat in our methodology, we aim to achieve natural, cohesive results that respect the unified anatomy of the face, thus avoiding compartmentalized approaches that might distort the natural facial structures.

#### Terminology: Replacing “midface” with “mesoface”

Minelli published an article questioning the traditional concept of malar fat compartmentalization in 2023.^7^ He described a locally thickened subcutaneous fat layer overlying the mimetic muscles, which he termed the “melo” fat pad. This concept challenges Owsley’s classical description of the malar fat, which was confined to the region above the zygomaticus major muscle.^12^ Minelli demonstrated that this fat extends inferiorly over the anterior buccinator, modiolus, depressor anguli oris (DAO), and upper platysma, continuing seamlessly into the jowl region. He argued that the term *melo* more accurately reflects the continuity of this fat beyond the malar area.

Based on our own anatomical dissections and clinical observations, we described similar findings in our previous publications^4^^,^^5^ and concur with Minelli’s conclusions. Another aspect of his work we commend is his detailed description of the superficial fat organized in a honeycomb-like pattern, delineated by a dense network of fibroelastic septa anchoring the skin to the underlying structures. He further distinguished the superficial from the deep fat by the orientation of these septa: perpendicular to the skin in the superficial layer and parallel to it in the deep layer. This structural organization has been corroborated through histological analysis, sheet plastination, and micro-CT imaging in another study by Minelli, in which he further challenged the classical concept of the SMAS within the midcheek region.^13^ We strongly support this view and consider the SMAS to be a discontinuous layer, whose morphology and presence vary according to the facial region. Therefore, it cannot be regarded as a constant or universally identifiable surgical plane. Each surgical approach should thus be tailored to the patient’s specific anatomy, addressing only the tissues that demonstrate true ptosis. The mesoface differs from Minelli’s melo fat pad, which represents a specific anatomical component.^7^ In contrast, the mesoface is a surgical concept that integrates multiple structures across different layers, each with distinct biomechanical behaviors. This approach allows for a tailored dissection zone in facelift surgery. While our concept builds upon the anatomical unity of the malar fat compartments described by Minelli, it extends beyond static anatomy to encompass the dynamic changes of facial aging, thereby providing a practical framework for facial lifting.

It is necessary to distinguish the mesoface from the “mobile SMAS unit” frequently described in deep-plane literature. We acknowledge that the concepts might overlap, particularly as both encompass the malar fat pad and its continuity with the jowl. However, we favor the term mesoface because “mobile SMAS” relies on an anatomical premise that may misrepresent the reality of the anterior midface. As noted by Minelli, the very existence of a continuous SMAS layer in this central area is questionable.^13^ More importantly, from a biomechanical perspective, the soft tissues extending from the malar prominence down to the jowl age and descend as a single, cohesive block. We argue that relying on classical SMAS subdivisions risks providing a fragmented and inaccurate understanding of these dynamic aging processes.

In our Conservation facelift, we embrace the term “mesoface,” as it encapsulates the unique anatomical and functional characteristics of the central facial area. We believe that a vertical subdivision of the face into thirds (from medial to lateral: proface, mesoface, and metaface) provides a more dynamic and anatomically relevant framework for understanding facial aging. This division allows consideration of the facial gliding planes during mimetic movements and their evolution over time.

Our observations suggest that sagging predominantly affects the mesoface, while the metaface and proface remain relatively stable and do not sag significantly. Therefore, the key to a natural and efficient facelift lies in repositioning the sagging tissues of the mesoface—namely the superficial malar fat and its jowl extension—while preserving the stable structures of the metaface.

In contrast, the traditional horizontal midface concept includes only the superficial malar fat, excludes the jowl, and overlaps with parts of the proface and metaface that are in fact stable.

### Application of mesoface concepts in conservation facelift

In the Conservation facelift technique, the mesoface represents a focal point of treatment due to its susceptibility to age-related ptosis, primarily affecting the skin and superficial fat layers. By concentrating on these elements rather than assuming distinct “compartments,” we follow an anatomically coherent approach that respects the continuity of the facial fat and soft tissue structure, as argued by Minnelli.^7^ This methodology allows for effective repositioning without creating artificial divisions in the facial anatomy, supporting our emphasis on natural, cohesive outcomes Most SMAS techniques are based on the anatomical description by Mitz and Peyronie.^14^ Traditional approaches, such as SMASectomy or SMAS plication, aim to achieve tightening of the SMAS over the parotid region.^15^ More advanced methods have introduced subsmas dissection with mobilization of the SMAS along specific vectors. Among these, two major techniques are widely recognized: the deep-plane facelift and the high-SMAS facelift.^16^ Both involve sub-SMAS dissection but differ in their point of entry: along the zygomatic arch with preauricular extension in the high-SMAS facelift, and along a line between the lateral canthus and mandibular angle in the deep-plane facelift according to Hamra,^17^ which is more anterior than in the high-SMAS technique. In contrast, our entry point is dynamic and patient-specific, defined strictly by the lateral border of the mesoface, which is frequently more anterior than Hamra's line.

Our approach differs from these established techniques in a fundamental way. All SMAS-based procedures involve mobilization of the SMAS in the metaface, a region where the SMAS is anatomically stable and does not undergo significant age-related sagging. In contrast, our technique addresses exclusively the mesoface, which is the only region demonstrably affected by gravitational descent with aging. As we have discussed, the superficial malar fat pad and its jowl extension represent the structures that truly sag with age. By selectively repositioning only these tissues, we avoid unnecessary manipulation of the metaface and thereby reduce the risk of creating unnatural or disharmonious results. Furthermore, this approach minimizes excessive traction on the tissues, which may account for the absence of neurapraxia in our series. Therefore, we avoid a one-fits-all strategy and instead provide a tailored approach for each patient by selectively repositioning sagging tissues. The conservation facelift share some similarities with SMAS-based procedures such as Baker’s lateral SMASectomy, such as the limited anterior release.^18^ However, the fundamental difference lies in tissue preservation. While the lateral SMASectomy relies on a strip of SMAS resection to generate lift and tension, our approach is strictly conservative, meaning no tissue is resected. Furthermore, we perform a limited deep plane dissection, right below the mesoface. Finally, the Conservation Facelift is distinct from Webster’s 'conservative facelift'.^19^ While they share similar terminology, we differ by elevating a longer subcutaneous flap (extending 2 to 3 cm beyond the mesoface's lateral border) and performing a true deep-plane entry, whereas Webster’s approach was limited strictly to SMAS plication.

Key differences between our Conservation facelift and other described techniques are highlighted in Table 1.

**Table 1:** Key differences between the Conservation facelift and other techniques.

	Deep plane entry point	Extent of dissection	Advantages	Drawbacks	Comments
Conservation facelift : our technique	Lateral border of the mesoface fat pad. It adapts to the patient facial anatomy	Limited undermining of the meso‑face fat pad (1–2 cm)	Safety due to limited undermining. Long lasting results. Addresses the mesoface and the neck	The learning curve for mesofacial border distinction in over-weight patients	Preservation of facial retaining ligaments.
Extended deep-plane lift	Lateral malar eminence to mandible angle, to anterior border of sterno-cleido-mastoid muscle	Often to the modiolus, above the Zygomaticus major belly	Cutaneous flap well vascularized. Long lasting results	Risk of nerve injury, Stensen’s duct injury. Long recovery	Retaining ligaments of the face are cut and repositioned by most surgeons.
Subperiosteal midface lift	Subperiosteal plane that can be accessed via the inferior palpebra or gingivolabial sulcus	Subperiosteal dissection over the whole maxilla.	Safety with the facial nerve. Near invisible scars.	Risk of infra-orbital nerve lesion. Addresses only the malar fat pad.de	No skin excision, just repositioning of the fat pads.

When learning or transitioning to this technique, emphasis is put on the pre-surgical markings as the entry point to the deep plane varies between each patient. Mesoface limits are easier to distinguish in older and thin patients. The mesoface lateral border should be identified pre-operatively with the patient standing and per-operatively during the subcutaneous plane dissection. Per-operatively the mesoface fat pad can be pulled with forceps to highlight the deep plane entry point. When entering the deep plane, we encourage dissection close to the deep border of the mesoface fat pad to avoid injury to deeper structures.

The main challenge of our technique is precisely defining the mesoface boundaries. We recommend starting with thin, older patients, where the mesoface is more distinct, and dissection follows a standard facelift approach with an intuitive deep plane entry. When confident, surgeons can transition to younger patients where distinction of the mesoface border is challenging. We don’t recommend our technique in over-weight patients as distinction between subcutaneous fat and the mesoface fat pad remains challenging. As a rule of thumb, initial patient selection should focus on individuals with a clearly defined mesofacial contour, where the indication is justified by the presence of sagging jowls and a BMI close to normal.

#### Preservation of the facial retaining ligaments

In recent years, studies and expert analyses have highlighted that successful facelift results can be achieved without cutting facial retaining ligaments, emphasizing safety and structural integrity in facial surgery.^20^, ^21^, ^22^ This approach is aligned with the work of Mendelson and other experts, who argue that ligaments serve as critical supports, maintaining facial stability and natural contour.^20^^,^^21^^,^^23^, ^24^, ^25^, ^26^ Cutting these ligaments can lead to structural imbalances, risking complications like sagging or asymmetry over time.^25^ Araghi advocates ligament-preserving techniques, suggesting repositioning, rather than severing, these ligaments allows for stable and long-lasting results.^21^^,^^25^ This approach is said to respect the natural “scaffold” of the face, where ligaments act as anchor points, enabling the lift to maintain facial harmony.^21^ Conversely, some surgeons contend that releasing the facial retaining ligaments, especially the zygomatic and masseteric, is essential to achieve adequate elevation of the midface.

In our experience, we believe preserving these ligaments reduces complications risk, such as nerve injury, and supports a natural postoperative appearance and quicker recovery.^27^^,^^28^ As previously described, we believe that only the ptotic tissues should be repositioned, thereby avoiding unnecessary disruption of the facial retaining ligaments. Although permanent injury to facial nerve branches is rare in deep plane facelifts, we observed no cases of temporary facial nerve paresis, compared to the 0.9% incidence reported in a large meta-analysis.^29^ This may be attributed to our limited dissection within the deep plane and selective repositioning of only sagging tissues, thereby reducing the risk of neurapraxia.

### Key factors in achieving successful facelift surgery

Medical techniques evolution reflects a continuous cycle of refinement and innovation, with facelift surgery remaining a cornerstone procedure in facial rejuvenation.

Recently, the “Deep Plane” facelift has been heralded as the gold standard in the field, endorsed by both practitioners and social media.^2^^,^^30^, ^31^, ^32^, ^33^ However, we propose a gentler, less invasive technique, inspired by dynamic analysis of facial aging, which preserves anatomical structures and minimizes tissue trauma, while achieving satisfying outcomes. We reported a hematoma rate of 4.3% and no cases of skin necrosis, which are comparable to or lower than the rates observed in standard deep plane facelifts (4.03% and 0.49%, respectively).^29^

Our Conservation facelift approach aligns with this ethos, offering a technique grounded in an anatomical, dynamic, and clinical understanding of age-related facial sagging.

### Limitations

We acknowledge that this article presents certain limitations. We introduce a new concept “the mesoface” based on observations of facial aging and anatomical dissections. The objective is to share the experience of the two senior authors and to illustrate how they implemented the mesoface concept in their deep plane facelift technique. As this is primarily a descriptive study, the absence of demographic data, standardized outcome measures, and long-term follow-up limits the level of evidence (Level V). Furthermore, it does not allow for direct comparisons with other facelift techniques because differences in patient populations may introduce confounding bias. The absence of validated outcome measures represents another limitation, as it reduces the objectivity of the assessment of harmonious and durable results and may introduce evaluator bias. In addition, revision surgery, touch-up procedures, and other secondary interventions were not systematically collected. Consequently, the long-term need for additional procedures could not be assessed, limiting the evaluation of the durability of the technique beyond the reported clinical outcomes. Finally, complication rates were assessed and reported by the operating surgeons rather than through independent external review, introducing the potential for observer bias. While the paper aims to provide an expert perspective on a modern facelift approach, further studies are needed to validate its advantages and to compare its effectiveness with existing techniques.

## Conclusion

The Conservation facelift is inspired by the natural dynamics of age-related descent. It aligns with a modern approach to facial rejuvenation that prioritizes both safety and long-term results through strategic repositioning of the skin and mesoface fat compartments. Clarifying the existence and functional role of the SMAS, still debated among experts, requires further research and may guide future refinements in facelift techniques.

## Patient consent

Written informed consent was obtained from all patients for the use of their clinical photographs in this publication.

## Ethical approval

Not applicable. This study adhered to the principles of the Declaration of Helsinki. All patient data were handled in compliance with applicable privacy standards, and written informed consent for the use of clinical information and images was obtained from all patients.

## Intellectual property notice

All concepts, and figures presented in this article are the exclusive intellectual property of Dr. Lakhdar Belhaouid and Dr. Pierre Quinodoz.

## Funding

None.

## Declaration of competing interest

None.

## Appendix Supplementary materials

Video 1 Mesoface delimitation video. Image, video 1 dummy alt text

Video 2 Conservation facelift technique video. Image, video 2 dummy alt text
